# Hemophagocytic Lymphohistiocytosis: An Unusual Presentation of Scrub Typhus

**DOI:** 10.7759/cureus.9390

**Published:** 2020-07-25

**Authors:** Navaneethakrishnan Suganthan, Menaka Mahakumara, Thaneswary Sooriyakumar

**Affiliations:** 1 Internal Medicine, University of Jaffna, Jaffna, LKA; 2 University Medical Unit, Jaffna Teaching Hospital, Jaffna, LKA; 3 General Medicine, University Medical Unit, Jaffna Teaching Hospital, Jaffna, LKA; 4 Hematology, Jaffna Teaching Hospital, Jaffna, LKA

**Keywords:** hemophagocytic lymphohistiocytosis (hlh), scrub typhus, pancytopenia

## Abstract

Scrub typhus is an important etiological cause for acute undifferentiated febrile illness in the Asia-Pacific region, including Sri Lanka. It is a mite-borne disease caused by Orientia tsutsugamushi. Hemophagocytic lymphohistiocytosis (HLH) is a rapidly progressive and potentially life-threatening hyperinflammatory syndrome rarely associated with scrub typhus. We herein describe a rare case of scrub typhus complicated by hemophagocytic lymphohistiocytosis in a 40-year-old previously healthy woman who presented with a history of an acute febrile illness. Following the observation of acute deterioration of hematological parameters despite the nature of the febrile illness, the rare association of hemophagocytic lymphohistiocytosis was considered, and this disease association was confirmed by fulfilling six out of eight of the diagnostic criteria of haemophagocytic lymphohistiocytosis. The patient made an uneventful recovery following treatment for the precipitating illness and with supportive care.

## Introduction

Scrub typhus is a mite-borne infectious disease, caused by Orientia tsutsugamushi, which is an obligate intracellular bacteria and is endemic in the Asia-Pacific region. The disease severity of scrub typhus can vary among individuals. Hemophagocytic lymphohistiocytosis is an aggressive and life-threatening condition of normal but overactive histiocytes and lymphocytes, which commonly appear in infancy, although it has been reported in all age groups. While primary hemophagocytic lymphohistiocytosis is an inherited form of syndrome and is a heterogeneous autosomal recessive disorder, secondary hemophagocytic lymphohistiocytosis occurs after strong immunologic activation, such as that which can occur with systemic infection (e.g., Epstein-Barr virus (EBV), dengue), immunodeficiency, or underlying malignancy [1-3]. Here, we describe a 40-year-old Sri Lankan woman presented with fever and pancytopenia. She was diagnosed with scrub typhus fever complicated with hemophagocytic lymphohistiocytosis, which is a rare association. She made an uneventful recovery with a course of antibiotic therapy and supportive care.

## Case presentation

A 40-year-old, previously healthy Sri Lankan woman was presented with a history of fever of 12 days duration, which was associated with myalgia, polyarthralgia involving large and small joints, malaise, and other constitutional symptoms. She also did admit to a history of headaches mainly confined to frontal areas without photophobia or symptoms of rhinosinusitis. She experienced nausea and vomiting for proceeding few days but denied diarrhea and abdominal pain. She had no symptoms that can localize the site of infection to either the respiratory or genito-urinary tract.

**Figure 1:**
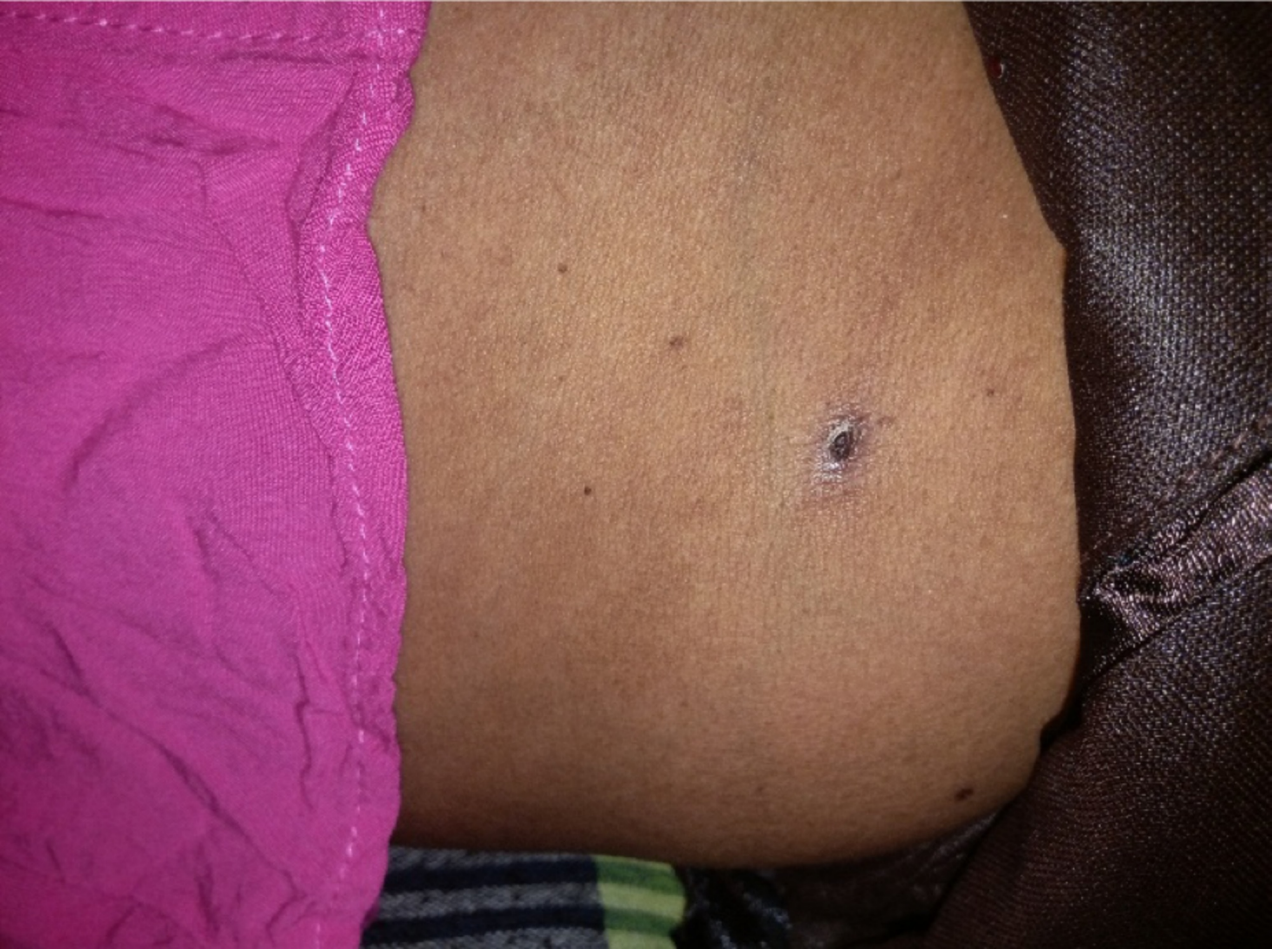
Eschar seen at right side inguinal region

On arrival, she was alert (Glasgow Coma Scale (GCS) 15) and recorded a high-grade fever (40.0°C). A general examination revealed pale conjunctivae and an eschar noted in the right inguinal area (Figure 1), with tender right inguinal lymphadenopathy. She had no neck stiffness or maculopapular rash. The initial cardiovascular assessment showed a blood pressure of 110/70 mmHg with a pulse rate of 120 regular beats per minute. The rest of the clinical examination was unremarkable. Based on the above clinical findings, she was diagnosed with scrub typhus.

Her initial investigations revealed pancytopenia, with a white blood cell (WBC) count of 1940/mm^3^, hemoglobin of 7.6 g/dL, and platelet count of 35,000/mm^3^. Inflammatory markers and hepatic transaminases were raised (C-reactive protein (CRP) 43.1 mg/L, erythrocyte sedimentation rate (ESR) 106 mm at the first hour, aspartate aminotransferase (AST) 537 U/L, alanine transaminase (ALT) 279 U/L). The rest of the basic biochemical profile, including renal function, showed no abnormalities (Table 1).

**Table 1:** Summary of results of blood investigations WBC: White blood cell, Hb: Hemoglobin, AST: Aspartate transaminase, ALT: Alanine transaminase

	Reference range	Day 12	Day13	Day 14	Day 15	Day 16	Day 17	Day 18	Review at 2 weeks
WBC /mm^3^	4000 - 11000	1,940	2,420	3,310	2,710	4,600	4,130	3,600	7200
Hb (g/dL)	11.8 -14.8	7.6	7.4	6.6	5.9	8.2 (post packed cell transfusion)	8.2	8.6	11.9
Platelets /mm^3^	150000-400000	35,000	23,000	30,000	28,000	58,000	91,000	124,000	321000
AST U/L	15 -37	537	367	232	156	98	75	48	29
ALT U/L	16 - 63	279	333	234	207	156	152	113	52

Mild splenomegaly was detected on an ultrasound scan of the abdomen with no other abnormalities. The indirect fluorescent antibody test for scrub typhus was performed, and it showed positive. Laboratory evaluation for dengue fever (nonstructural protein 1 (NS1) antigen, immunoglobulin M (IgM), and IgG antibody for dengue) came negative. The blood picture showed severe neutropenia, thrombocytopenia with severe anemia, with no evidence of disseminated intravascular coagulation or abnormal cells.

**Figure 2:**
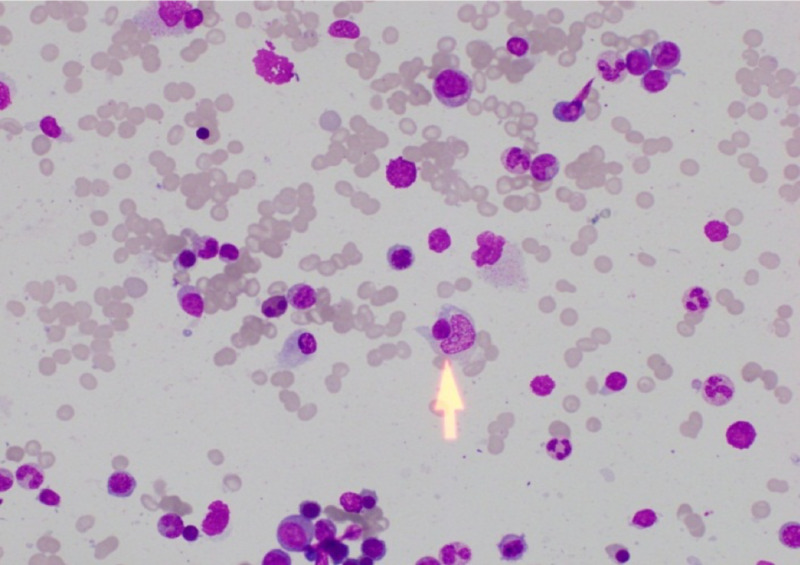
High-power view of bone marrow aspirate showing an increased number of histocytes and hemophagocytosis (arrowhead)

On observation of progressively worsening pancytopenia, the rare association of hemophagocytic lymphohistiocytosis was considered. Further investigations revealed serum ferritin of 725 ng/mL and isolated hypertriglyceridemia of 3.72 mmol/L. Subsequently, she underwent bone marrow aspiration, which showed an increased number of histocytes and hemophagocytosis and these findings were compatible with a diagnosis of hemophagocytic lymphohistiocytosis likely to be secondary to underlying infection (Figure 2). As she was fulfilling six out of eight criteria for the diagnosis of hemophagocytic lymphohistiocytosis, the rare association of hemophagocytic syndrome triggered by scrub typhus was made. Further, other common precipitating causes for hemophagocytic syndrome such as Epstein-Barr virus (EBV) and cytomegalovirus (CMV) infections were excluded by negative antibody tests.

The patient was commenced on doxycycline along with other supportive care, including packed cell transfusion. After three days of antibiotic treatment, progressive pancytopenia started to show signs of improvement in all three cell lines. On Day 6 following admission, she was sent home with hemoglobin of 8.6 g/dL, platelet of 124,000/mm^3^, and white blood cells of 3,600/mm^3^. On review in two weeks, she was asymptomatic and her biochemical and hematological profiles were back to normal as a marker of uneventful recovery.

## Discussion

Scrub typhus is a mite-borne infectious disease caused by Orientia tsutsugamushi [1]. Scrub typhus is endemic to a part of the world known as the ‘tsutsugamushi triangle,’ which extends from northern Japan and far-eastern Russia in the North, to northern Australia in the South, and to Pakistan in the west [4]. Diagnosing scrub typhus early in its course can be difficult because many conditions can present with a high fever; however, the presentation of the rash, a history of exposure to endemic areas, and the presentation of the sore caused by the bite can be diagnostic [2]. The mortality of typhus varies from 1% to 60%, depending on the pathogenic strain and the geographic area.

Hemophagocytic lymphohistiocytosis is a syndrome of excessive immune activation, and it is aggressive and life-threatening in nature. These patients might have already had a prolonged hospitalization or deterioration of clinical condition without having a clear diagnosis before the possibility of the hemophagocytic syndrome is raised. Many patients with hemophagocytic lymphohistiocytosis have a predisposing genetic defect, and/or an immunologic trigger, which can include infection, malignancy, a rheumatologic disorder, such as juvenile idiopathic arthritis, or another disorder associated with immune dysregulation [1].

According to the literature review, there are a few cases reported worldwide where hemophagocytosis was triggered by scrub typhus, mainly from India, China, Japan, and South Korea [5-8]. The only case report published from Sri Lanka was in 2009 [9]. We are reporting a second case scenario with a similar relationship, revealing the importance of this association.

The differential diagnosis of this syndrome is considered as several multisystem illnesses, which are characterized by fever, neurologic symptoms, and hepatic failure [1]. The diagnostic criteria of the hemophagocytic syndrome are fever >38.50°C, splenomegaly, peripheral blood cytopenia, hypertriglyceridemia and/or hypofibrinogenemia, hemophagocytosis in bone marrow, spleen, lymph node, or liver, low or absent natural killer (NK) cell activity, ferritin > 500 ng/ml, and elevated soluble CD25. Out of these criteria, there should be a minimum of five criteria to make the diagnosis, and our patient fulfilled six out of eight criteria, and, therefore, the diagnosis was made.

Based on the fulfillment of diagnostic criteria or a high clinical suspicion of hemophagocytic lymphohistiocytosis, treatment should be started urgently. Untreated patients with hemophagocytic lymphohistiocytosis have survival of months, due to progressive multiorgan failure [3]. The treatment of haemophagocytic lymphohistiocytosis includes etoposide, dexamethasone, methotrexate, hematopoietic stem cell transplantation, and transfusions of plasma products as needed in acutely ill/deteriorating patients, as well as treating the triggering condition such as an infection [3]. As our patient’s clinical condition was stable, she was treated with doxycycline along with other supportive care, including packed cell transfusion. She made an uneventful recovery.

## Conclusions

Early diagnosis of scrub typhus in endemic areas is essential for the initiation of specific antibiotics, to prevent complications. History, a thorough clinical examination, as well as strong clinical suspicion, is needed to diagnose scrub typhus in a setting where antibody testing is not freely available. When there is a rapid deterioration of a febrile illness with dropping blood counts, hemophagocytic lymphohistiocytosis should be considered, as prompt diagnosis and early treatment reduce the mortality associated with this condition.
